# Significance of monocyte infiltration in patients with gastric cancer: A combined study based on single cell sequencing and TCGA

**DOI:** 10.3389/fonc.2022.1001307

**Published:** 2022-11-21

**Authors:** Wei Xu, Dongxu Zhao, Xiaowei Huang, Man Zhang, Wenxin Zhu, Chunfang Xu

**Affiliations:** ^1^ Department of Gastroenterology, The First Affiliated Hospital of Soochow University, Suzhou, Jiangsu, China; ^2^ Department of Interventional Radiology, The First Affiliated Hospital of Soochow University, Suzhou, Jiangsu, China; ^3^ Department of General Surgery, The First Affiliated Hospital of Soochow University, Suzhou, Jiangsu, China; ^4^ Department of Emergency Medicine, The Affiliated Hospital of Xuzhou Medical University, Xuzhou, Jiangsu, China; ^5^ Department of Gastroenterology, Kunshan Third People’s Hospital, Suzhou, Jiangsu, China

**Keywords:** gastric cancer, monocytes, single cell sequencing, tumor immune microenvironment, prognosis, tumor therapy.

## Abstract

**Background:**

Gastric cancer is still one of the most lethal tumor diseases in the world. Despite some improvements, the prognosis of patients with gastric cancer is still not accurately predicted.

**Methods:**

Based on single cell sequencing data, we conducted a detailed analysis of gastric cancer patients and normal tissues to determine the role of monocytes in the progression of gastric cancer. WCGA facilitated our search for Grade-related genes in TCGA. Then, according to the marker genes and cell differentiation genes of monocytes, we determined the cancer-promoting genes of monocytes. Based on LASSO regression, we established a prognostic model using TCGA database. The accuracy of the model was verified by PCA, ROC curve, survival analysis and prognostic analysis. Finally, we evaluated the significance of the model in clinical diagnosis and treatment by observing drug sensitivity, immune microenvironment and immune checkpoint expression in patients with different risk groups.

**Results:**

Monocytes were poorly differentiated in tumor microenvironment. It mainly played a role in promoting cancer in two ways. One was to promote tumor progression indirectly by interacting with other tumor stromal cells. The other was to directly connect with tumor cells through the MIF and TNF pathway to play a tumor-promoting role. The former was more important in these two ways. A total of 292 monocyte tumor-promoting genes were obtained, and 12 genes were finally included in the construction of the prognosis model. A variety of validation methods showed that our model had an accurate prediction ability. Drug sensitivity analysis could provide guidance for clinical medication of patients. The results of immune microenvironment and immune checkpoint also indicated the reasons for poor prognosis of high-risk patients.

**Conclusion:**

In conclusion, we provided a 12-gene risk score formula and nomogram for gastric cancer patients to assist clinical drug therapy and prognosis prediction. This model had good accuracy and clinical significance.

## Introduction

Gastric cancer is one of the most common types of cancer worldwide. As of 2021, stomach cancer was ranked fifth overall and fourth in terms of death (1–3). Most gastric cancer (about 90%) is adenocarcinoma, which originates from the most superficial or mucosal glands of the stomach. The histological subtypes of gastric cancer are generally separated into intestinal and diffuse subtypes (4). According to the location of stomach cancer, some researchers classify it into cardia gastric cancer and non-cardia gastric cancer (5, 6). Helicobacter pylori infection, alcohol intake, smoking, and low fruit intake are considered to be important pathogenic factors for gastric cancer (7–9). Helicobacter pylori infection is verified to be the most important one of these (10). Correa et al. pointed out that the process from normal gastric epithelium to gastric cancer was a multi-stage and multi-factor process. Strong acidic stimulation environment, direct damage of Helicobacter pylori to gastric epithelium, and the decomposition of nitrate in food by HP into nitrite or n-nitroso compounds played a crucial role in this process (11, 12). There are considerable gender and regional disparities in the incidence of stomach cancer. Gastric cancer is twice as common in men as it is in women, according to reports, and the incidence in North America and Europe is significantly lower than that in Asia (13). The prevalence of cancer screening and the improvement of food hygiene have made the incidence and mortality of gastric cancer gradually decline in the past half century. But in recent years, due to improper use of antibiotics and acid inhibitors, the gastric microbial environment has been destroyed, and the incidence of gastric cancer has rebounded in some countries (14, 15). The prognosis of gastric cancer patients sometimes varies substantially due to the significant variability across gastric cancer tissues (16). While the TNM staging system and Borrmann classification are frequently used to assess the prognosis of gastric cancer patients, their predictive accuracy is not sufficient (17). Therefore, exploring the sources of gastric cancer heterogeneity and finding an effective prediction method to guide the clinical diagnosis and treatment of gastric cancer patients are still important goals in gastric cancer research.

Tumor immune microenvironment is a unique barrier formed of tumor cells, surrounding immune cells, fibroblasts, and immunological factors released by these cells. Its purpose is to block tumor immunity and encourage tumor proliferation and spread (18). Interaction between tumor cells and microenvironment is one of the reasons for tumor heterogeneity (19). Monocytes are one of the important components of tumor microenvironment. Under normal circumstances, monocytes can flow with blood or stay in tissues, play a role in phagocytosis and killing microorganisms and tumors, and are the intermediary connecting innate immunity and adaptive immunity. In the tumor microenvironment, monocytes are recruited by tumor tissues and become their accomplices. A large part of monocytes will be further differentiated into M2 macrophages (tumor-associated macrophages), which inhibit the function of peripheral immune cells and promote tumor immune escape. The other portion enhances Treg cell aggregation directly, inhibits CD4+/CD8+ T cell antitumor activity, and promotes vascular survival and extracellular matrix transformation (20–24). Therefore, the infiltration of monocytes in tumor microenvironment promotes tumor progression, causes tumor heterogeneity, which is one of the important reasons for the failure of tumor treatment.

Traditional high-throughput sequencing requires a large number of cells to obtain enough DNA for sequencing due to technical restrictions. As a result, the sequencing data represent the ‘ensemble’ gene expression of these cells, disregarding the specific characteristics and roles of individual cells. We have been able to perform high-throughput sequencing analysis of genomes, transcriptomes, and epigenetics at the single cell level, revealing the gene structure and gene expression status of a single cell and reflecting heterogeneity between cells, thanks to the development of next-generation sequencing (NGS) and third-generation sequencing (TGS) technologies (25–27). Several previous studies have carried out single-cell sequencing analysis on human gastric cancer tissues (28–30). As a result, we conducted in-depth studies on the significance of monocyte infiltration in gastric cancer tissues, searched for potential monocyte oncogenes in gastric cancer tissues, and explored the functional status of monocytes at different stages and pathways of action with other cells, all based on published single-cell sequencing data.

## Materials and methods

### Download and collation of single cell data

Single cell data used in this study came from GEO database, registration number was GSE163558. The original data included 3 cases of primary gastric cancer, 1 case of peritumoral normal tissue and 6 incidences of metastatic stomach cancer. We chose three examples of primary gastric cancer tissue for analysis and 1 case of normal tissue for subsequent data analysis. Single cell data has been preprocessed by CellRanger (10X Genomics) (version 3.0.2). Cells whose genes were less than 200 or more than 5000 were filtered out. Cells with more than 20% mitochondrial DNA were also excluded.

The remaining cells’ gene expression matrix was normalized using a global-scaling approach with a default scale factor and a natural-log transformation followed by a log1p transformation. Finally, the normalized expression was scaled using the ScaleData method to eliminate unneeded mutation sources.

In order to prevent low-quality cell residues after previous data preprocessing, thereby affecting the accuracy of downstream analysis. Therefore, on the basis of previous preliminary data filtering, we integrated and further controlled the single cell data based on the “Seurat” package of R (version 4.12) (version 4.1.0): (1) We excluded cells with less than 500 or more than 6000 gene expression. (2) The UMI count value of each cell sequencing must be greater than 1000, and the top 3% cells with the largest UMI count value were excluded. (3) The proportion of mitochondrial gene expression in each cell in the total gene should be less than 35%, and the first 2% cells with the highest mitochondrial gene expression should be removed. (4) Calculate the proportion of rRNA expression in the total gene and remove the smallest and largest proportion of the first 1% cells.

After acquiring high-quality cells, we utilized the ‘NormalizeData’ function to divide each gene’s expression in each cell by the total expression and multiply by the scale factor 10000. Then we normalized it by log to eliminate possible technical errors. Finally, we used the ‘FindVariableFeatures’ function to search for highly variable genes (HVGs), which expressed differently in different cells. We chose the first 2000 genes for downstream analysis.

### Annotation of cell types and screening of key subgroups

PCA is a technique for linear dimension reduction. The high-dimensional data are projected to a low-dimensional space using a linear transformation, and the data variance is predicted to be greatest in the projected dimension. This way, we can use fewer data dimensions while retaining the properties of a greater number of original data points. TSNE is a nonlinear dimension reduction technique. TSNE transforms the distance between two points in a high-dimensional space to their likelihood of resemblance in a low-dimensional space, while preserving the minimal sum of the conditional probability difference between the two points in high-dimensional and low-dimensional space. In addition, the long tail of the t-distribution is used to solve the overlap problem when high-dimensional data is mapped to low-dimensional data, which leads to a better chance of selecting related objects. We used both dimensionality reduction methods simultaneously to make the results of cluster analysis more reliable.

After clustering analysis, the ‘FindAllMarkers’ function was used to identify the marker genes for each subgroup (the filtering standard was logFC > 1, P< 0.05). After getting the marker genes for each subgroup, we annotated the cell type using the HumanPrimaryCellAtlasData database in the ‘SingleR’ package (version 1.8.1), and then validated the annotation results on the CellMarker website (31) to ensure the accuracy of the annotation. Finally, we used the FindAllMarkers function again to find the marker genes of annotated cells.

By using a two-sided test, Fisher’s exact test was utilized to determine whether there was a significant difference in the cluster of cells between tumor and normal samples. The screening criteria were P<0.05 and FC>4 or FC<0.25. The analyse_sc_clusters function in the ReactomeGSA ‘package (version 1.8.0) was used for enrichment analysis of the function of cell clusters. Subsequently, the difference between the highest and minimum values of each pathway score was determined. Then we sorted the expression of each pathway according to the difference. In the end, the first 10 pathways with the largest difference in pathway score were extracted through the pathways function as the results of pathway enrichment.

### Pseudotime analysis and cell communication analysis

We used the ‘monocle’ package (version 2.22.0) to perform pseudotime analysis on single cell data. Pseudotime analysis is one of the methods of trajectory analysis. According to the temporal gene expression of each cell, each cell could be arranged in the corresponding trajectory according to the quasi-time. The samples were classified into cell groups based on the expression of genes in multiple differentiation states. The intuitive pedigree tree diagram could be generated to predict cell differentiation and development trajectory. In the first place, we extracted the key cell subgroups and selected their marker genes for the next analysis. Subsequently, we used the DDRTree method of reduceDimension function to reduce the dimension of the data, calculated the development time, inferred the trajectory, and sorted the cells according to the quasi-time sequence to visualize the results. The emergence of branch points represented the programmed changes of cells, such as cell fate differentiation. The statistical method of beam was used to analyze the cell data and designated nodes after quasi-time sorting. The contribution value of genes in the process of cell development was calculated, and then the differential genes that played a critical role in the development and differentiation of cells were screened.

Cell communication referred to the information sent by one cell through the medium to another cell to produce corresponding reactions. Intercellular communication through chemical signaling molecules is the most commonly used communication mode in animals and plants. Cell-cell communication mediated by ligand-receptor complexes was critical in coordinating many biological processes such as development, differentiation, and inflammation. The package ‘CellChat’ (version 1.1.3) was used to infer and evaluate the cell-cell interaction network. We began by identifying overexpressed ligands or receptors within a cell group and then projected the gene expression data onto the protein-protein interaction (PPI) network. If either the ligand or the receptor is overexpressed, the interaction between the overexpressed ligand and receptor is recognized. Following that, we calculated the probability of communication at the signaling route level by computing the probability of communication for all ligand-receptor interactions associated with each signaling pathway, and calculated the aggregated communication network between cells by calculating the number of links or summarizing the communication probability. Finally, we established a cell communication network at the cell ligand-receptor and signaling pathway level. In additionally, we analyzed and visualized the communication network in which monocytes participate and contribute greatly.

### Download and collation of transcriptome data

The data of training set were from the TGCA database. We downloaded the transcriptomes of patients with and without stomach cancer, retrieved the raw data for integration, and annotated the genes. For repeated expression genes, we take the average of their expression. Finally, we got the gene expression matrix of TCGA cohort. We sorted out the clinical data, extracted TCGA number, survival time (day), survival status (survival or death), gender, age, tumor stage, T, N, M and grade. The test set came from the GEO database with the registration number GSM2235556, including sequencing data for 76 primary gastric cancer tissues. The transcriptome data were collated and annotated. In clinical data, we only focused on the survival time and survival status of patients. Gene mutation data came from TCGA database, and the data type was ‘Masked Somatic Mutation’. Finally, the study collected data on 434 mutations.

### Weighted gene co-expression network analysis

In the TCGA cohort, weighted gene co-expression network analysis (WGCNA) was utilized to uncover genes associated with tumor grade. WGCNA is a method for assessing gene expression patterns in numerous samples that may cluster genes with similar expression patterns and analyze the association between modules and specific phenotypes. The ‘WGCNA’ package served as the foundation for WGCNA (version 1.70.3). To make the connection between genes in the network obey the scale-free network distribution, we calculated the correlation coefficient between any two genes and took the weighted value of the correlation coefficient (i.e., the Nth power of the gene correlation coefficient). This could transform the adjacency matrix into the Tom matrix. Then, using weighted correlation and a specified standard, we did hierarchical clustering analysis and segmented the clustering findings to obtain various gene modules, which were represented by the branches of the clustering tree and different colors. Finally, we assessed the association between gene modules and clinical case features, selected the modules associated with Grade, and retrieved the genes associated with those modules for further investigation.

### Acquisition of potential oncogenes from monocytes

We obtained the marker genes and cell cycle-related genes of monocytes through the analysis of single cell data, and obtained the grade-related genes in the TCGA cohort through WGCNA. Cell cycle-related genes and grade-related genes were among those found to be closely linked to cell development and differentiation, which might be an important potential oncogene for gastric cancer. We discovered monocyte-specific differentiation-related genes by intersecting the three. These genes regulated monocyte growth and differentiation, which could be crucial in the advancement of gastric cancer. As a result, we created a prognostic model based on these genes to test their capacity to predict outcomes.

### Construction and validation of prognostic model

The gene expression of monocyte potential oncogenes in TCGA cohort was extracted, and the difference was analyzed based on ‘limma’ package (version 3.50.1) (P< 0.05, logFC = 1, that is, the difference multiple was 2 times). Following that, clinical data was integrated with the expression levels of differential genes in the TCGA and GEO cohorts (survival time, survival status). Univariate COX regression analysis was used to evaluate prognostic genes in differentially expressed genes (DGEs) using the ‘survival’ package (version 3.2.13). A prognostic prediction model was built using the Least absolute shrinkage and selection operator (LASSO) from the glmnet package (version 4.1.3). LASSO is a linear regression method that uses L1-regularization to make portion of the learned feature weights 0 in order to achieve the goal of sparse and feature selection. It could not only keep the model from being too good in the training samples, resulting in poor performance in the validation and test data sets (i.e., overfitting), but it could also keep the model from having too many variables (better than Ridge regression).

We used a range of test methodologies to determine the prognosis model’s prediction performance. Principal component analysis (PCA) is a dimension reduction method that converts highly correlated attributes/variables in the data into independent or irrelevant new attributes/variables, which uses fewer new attributes/variables (principal components) to explain most of the attributes/variables in the original data. The receiver operating characteristic curve (ROC) is a graphical representation of the continuous variable sensitivity and specificity. It calculates a series of sensitivity and specificity for continuous variables by setting many distinct thresholds, and then draws a curve with sensitivity as the ordinate and (1specificity) as the abscissa. The greater the area under the curve, the more precise the diagnosis. Independent prognosis analysis was performed to determine whether the prognostic model could be employed independently of other clinical characteristics as prognostic factors. The evaluation methodologies used were COX regression analysis on a single component and COX regression analysis on multiple factors. Additionally, we performed survival analyses on the overall survival (OS) and progression-free survival (PFS) of patients classified into distinct risk groups based on their risk score. The data were visualized using the Kaplan-Meier curve. Finally, we assessed the expression of prognostic model genes in normal and gastric cancer tissues from the human protein atlas (HPA) database.

### Clinical relevance, immune microenvironment, gene mutation and drug treatment sensitivity

We examined the clinical pathological features, immune microenvironment status, and response to pharmacological treatment of patients in various risk groups using the risk score algorithm. We first determined if there were statistically significant variations in the age, gender, Stage, T, N, M, and Grade of patients in various risk categories, and then visualized the data using a box plot. The CIBERSORT algorithm was used to analyze immune infiltration in patients belonging to various risk groups. CIBERSORT is a linear support vector regression-based technique for deconvolution of the expression matrix of human immune cell subtypes. By default, this approach produced the gene expression feature set for 22 immune cell subtypes (LM22) based on the known reference data set. The sensitivity analysis of drugs was performed using the R package ‘pRRophetic’ (version 0.5) developed by Paul Geeleher in 2014 (32). The pRRophetic technique developed a ridge regression model based on the expression profiles of the GDSC cell line and the TCGA gene to forecast drug half inhibitory concentrations (IC50, the corresponding drug concentration when the ratio of apoptotic cells to total cells was equal to 50%). The results of drug sensitivity analysis were shown by box plot and correlation. The box plot was the distribution of IC50 between different samples, and the correlation was the relationship between model score and IC50. Finally, we did gene mutation analysis on the genes associated with the prognosis model, assessed gene graph mutation and co-mutation relationships, and identified genes with the highest mutation frequency in various risk groups.

### Variation analysis of gene sets, enrichment analysis, and protein interaction analysis

Gene set variation analysis (GSVA) is a non-parametric and unsupervised algorithm. We used the ‘GSVA’ package (version 1.42.0) to estimate the kernel density of the expression data (used to estimate the unknown density function in the probability theory, which is one of the nonparametric test methods). Subsequently we ranked the expression levels of the samples based on the results of the kernel density estimation. Each gene set was calculated for the rank statistics similar to the K-S test, and finally obtained the enrichment score. The genes of patients in different risk groups were analyzed again for GO and KEGG analysis. Both GO and KEGG analyses were performed using the package ‘org.Hs.eg.db’ (version 3.14.0). GO analysis was used to classify genes according to their molecular function, cellular component, and biological process. KEGG is the most extensively used publicly accessible database of metabolic pathways. Finally, we utilized the STRING database to analyze the protein interaction networks (PPI) of differentially expressed genes and the ‘Cytoscape’ software (version 3.9.1) to show the analysis results, seeking for core genes in a large number of differentially expressed genes (minimum required interaction score = 0.7).

### The website of database

GEO: https://www.ncbi.nlm.nih.gov/geo/

TCGA: https://www.cancer.gov/about-nci/organization/ccg/research/structural-genomics/tcga

CellMarker: http://bio-bigdata.hrbmu.edu.cn/CellMarker/

STRING: https://cn.string-db.org/

HPA: https://www.proteinatlas.org/

## Results

### The immune infiltration analysis of TCGA and single cell sequencing analysis showed that monocytes were crucial infiltrating cells in gastric cancer tissues

**Figure 1:**
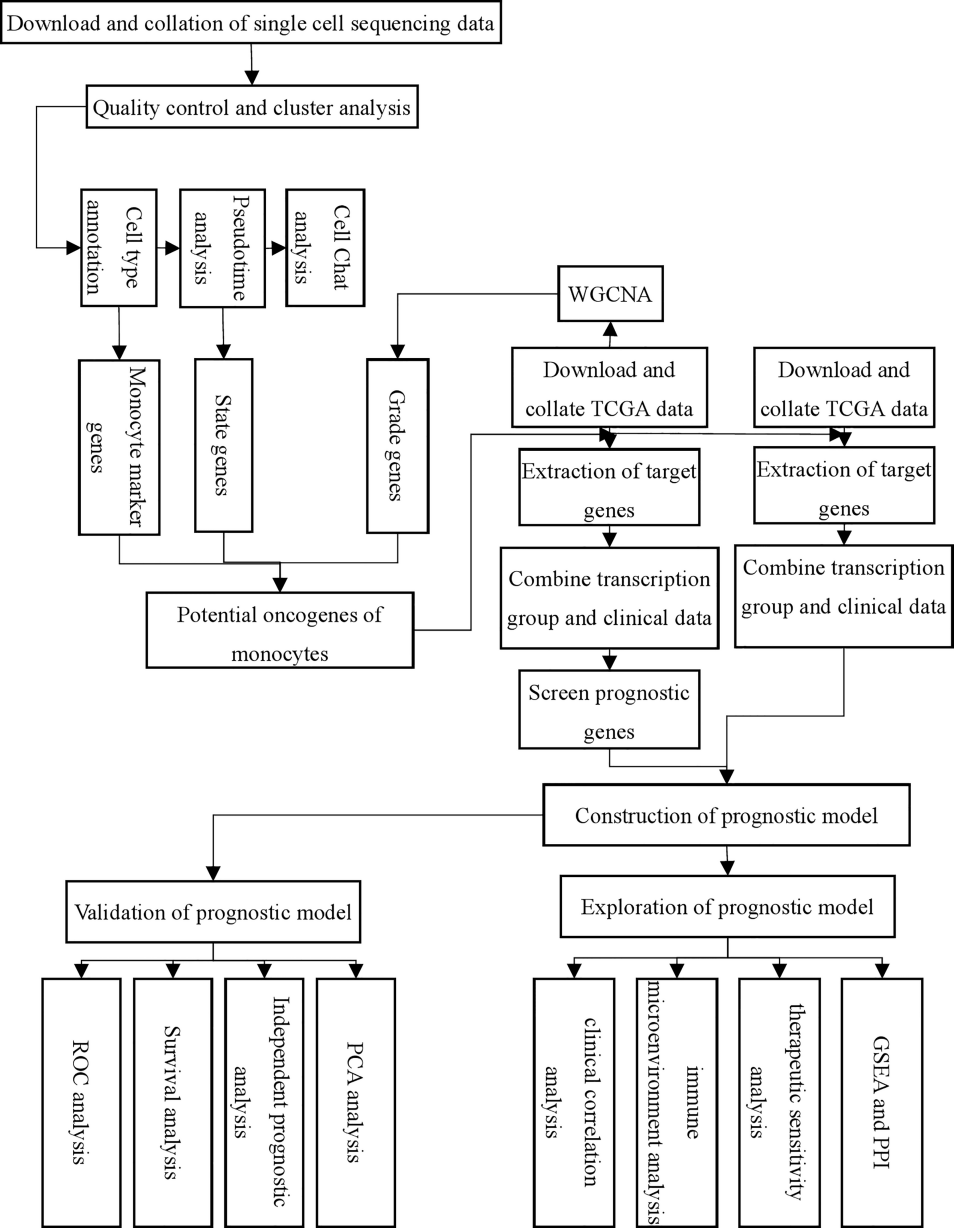
Flow chart of this study.

**Figure 2:**
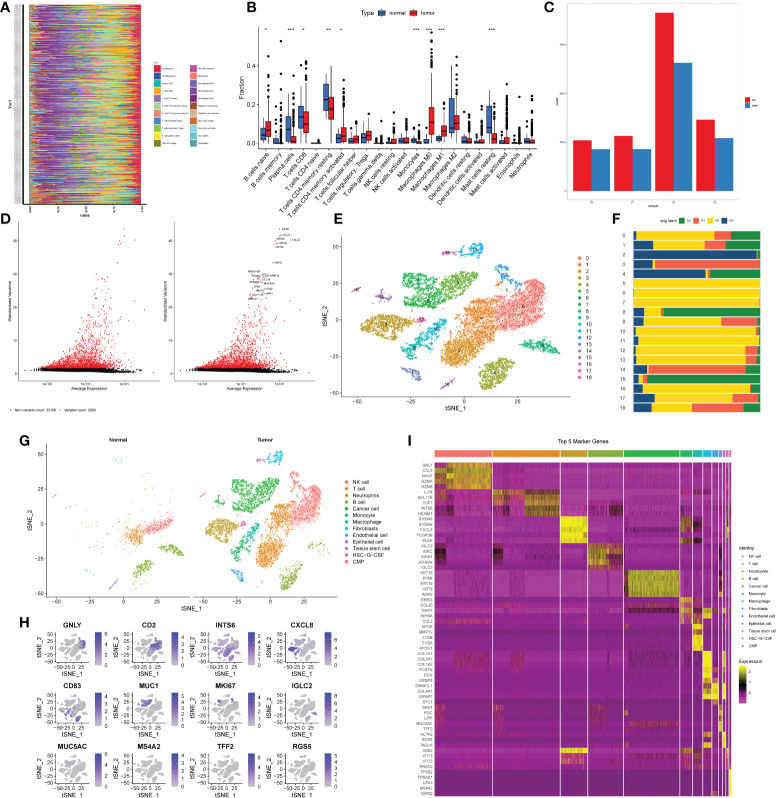
Processing of single cell sequencing data from GEO and annotation of cell types. **(A, B)** Results of immune cell infiltration analysis of gastric cancer patients and normal patients based on CIBERSORT algorithm in TCGA database. **(A)** Immunocyte infiltration heatmap. The longitudinal axis was the ID of the patient, the tumor patient above the dashed line, and the non-tumor patient below the dashed line. **(B)** Box plot. The horizontal axis was immune cells, red represented tumor patients, and blue represented non-tumor patients. *P< 0.05, **P< 0.01, ***P< 0.001. **(C)**. Bar plot of cell number changes in 3 tumor samples and 1 non-tumor sample after quality control. Red was the quantity before quality control, blue is the quantity after quality control. **(D)** High-variable genes in single-cell transcriptome genes. The black dots indicated that there was no significant difference in the expression of genes among cells, and the red dots indicated that the expression of genes was significantly changed among cells. The right figure showed the names of the top 20 hypervariable genes. **(E)** The results of TSNE cluster analysis. **(F)** Proportion of each cluster in tumor tissues and non-tumor tissues. **(G)** The results of cell type annotation for each cluster. The left and right parts represented the cell type annotation results of non-tumor and tumor tissues respectively. **(H, I)** Visualization results of marker genes. **(H)** The heatmap of the first five marker genes of each cell subgroup. The horizontal axis was the cell name, and the vertical axis was the gene name. Yellow indicated high gene expression, and purple indicated low gene expression. **(I)** Expression of single marker gene in cells. Purple indicated high expression, and gray indicates\d low expression.

The results of the TCGA clinical data analysis are summarized in **Table 1**. The study’s flow chart is depicted in **Figure 1**. The immune infiltration analysis of TCGA cohort showed that the number of monocytes in gastric cancer tissues was significantly different from that in normal tissues (**Figures 2A** **, B**). Subsequently, we aimed to verify it in single cell sequencing data. After strict quality control, 13588 cells and 24159 non-repeated genes were finally identified (**Figures 2C** **, D**). After PCA dimensionality reduction and TSNE clustering, 18 clusters (**Figures 2E** **, F**) were obtained. **Supplementary Table 1** as a supplement to **Figure 2F** showed in detail the proportion of each cell cluster in different samples. We annotated these 18 clusters through marker genes, and finally obtained 12 cell subgroups. According to the difference of cell-derived tissues, all cells were divided into tumor tissue origin and normal tissue origin (**Figure 2G**). The marker genes of each cell subgroup were displayed in the form of heatmap and distribution map (**Figures 2H** **, I**). **Supplementary Table 2** showed the marker genes of all cells as a supplement to **Figures 2H–I**. After differential analysis, we found nine distinct cell subgroups that were differentially expressed in tumor and normal tissues: Neutrophils, B cell, Cancer cell, Monocyte, Macrophage, Fibroblasts, Endothelial cells, Epithelial cells, and HSC-G-CSF. Thus, we established that monocytes infiltrated tumor and normal tissues differently and are critical infiltrating cells in gastric cancer tissues.

**Table 1:** Clinical data of TCGA patients.

		Number	Percent
Age	<=65	197	0.44
	>65	241	0.54
	unknow	5	0.01
Gender	MALE	285	0.64
	FEMALE	158	0.36
	unknow	0	0.00
Grade	G1	12	0.03
	G2	159	0.36
	G3	263	0.59
	unknow	9	0.02
Stage	Stage I	59	0.13
	Stage II	130	0.29
	Stage III	183	0.41
	Stage IV	44	0.10
	unknow	27	0.06
T	T1	23	0.05
	T2	93	0.21
	T3	198	0.45
	T4	119	0.27
	unknow	10	0.02
N	N0	132	0.30
	N1	119	0.27
	N2	85	0.19
	N3	88	0.20
	unknow	19	0.04
M	M0	391	0.88
	M1	30	0.07
	unknow	22	0.05

### Monocyte growth and differentiation in gastric cancer tissues, as well as the level of signaling pathway expression

**Figure 3:**
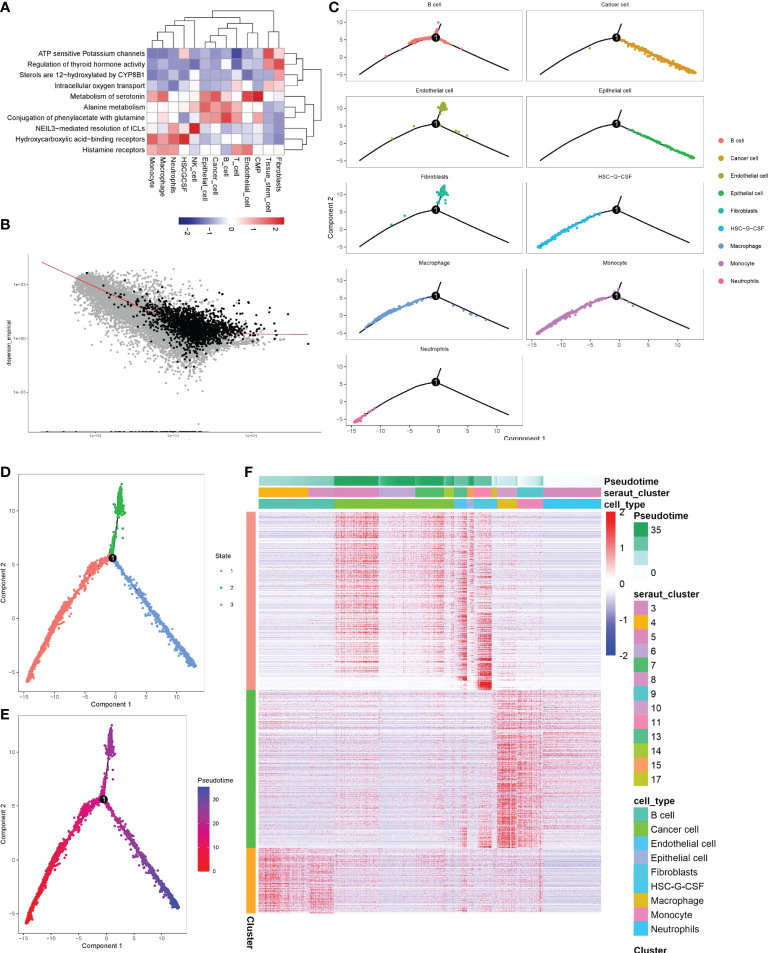
Identification of the significance of monocytes in gastric cancer by screening key clusters and pseudotime analysis. **(A)** Enrichment analysis of key cell clusters and cell function in gastric cancer. Red means function up, blue means function down. **(B)** Using pseudotime analysis to determine the genes related to cell differentiation (black representation). **(C–F)** The visualization of the pseudotime analysis. The line was the evolutionary trajectory. Each point represents a cell, and the occurrence of branch nodes represented a procedural change in the cell. **(C)** Differentiation trajectory of key cell clusters in pseudotime analysis. **(D)** Pseudotime analysis divided all cells into three branches, different colors represented different cell clusters. **(E)** All cells were sorted by color from deep to shallow within the pseudotime. **(F)** Heatmap of differentiation-related genes determined by beam algorithm. It mainly represented the distribution of differentiation-related genes of each cell subgroup in different branches, and the longitudinal axis represented the differential genes of the three branches. The longitudinal axis mainly represented the name of each cell subgroup. Red indicated high gene expression.

To advance research on the involvement of monocytes in stomach cancer, we performed ReactomeGSA functional enrichment analysis of key subgroups. The results showed that Histamine receptors, Hydroxycarboxylic acid-binding receptors, NEIL3-mediated resolution of ICLs, Metabolism of serotonin and other signaling pathways were significantly up-regulated in monocytes. ATP-sensitive potassium channels, Regulation of thyroid hormone activity, Sterols are 12-hydroxyated by CYP8B1, Intracellular oxygen transport and other signaling pathways were significantly down-regulated (**Figure 3A**), suggesting that a variety of functions of tumor-infiltrating monocytes were significantly altered.

By screening 10722 marker genes, 1801 genes were included in the pseudotime (**Figure 3B**). pseudotime analysis revealed the differentiation trajectory of monocytes (**Figure 3C**). Most monocytes were in cluster1 stage, with a high degree of differentiation, and a small part was in cluster3, with a low degree of differentiation (**Figures 3D** **, E**). In addition, we also found that tumor cells mainly originated from gastric epithelial cells and were in a high differentiation stage (**Figure 3C**). Then we found 1798 genes that gradually increased or decreased with the differentiation time by Beam analysis, these genes were related to cell state differentiation (**Figure 3F**).

**Figure 4:**
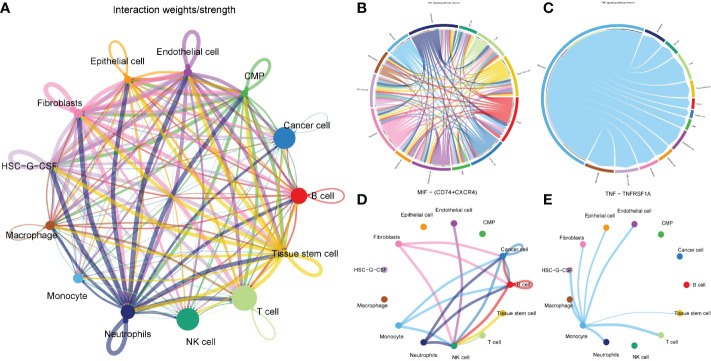
Cell communication analysis to determine the action network of monocytes in tumor microenvironment. **(A)** Network diagram of interaction of all cells in gastric cancer tissue. Each connection represented a connection, and the line’ s thickness represents the strength of the connection. Each point represented a cell subgroup, and the size of the point represented the weight of this cell subgroup in the network. **(B, C)** Signaling pathway that monocytes directly interacted with tumor cells. **(D, E)** The two pairs of ligand-receptors that contributed most to the MIF and TNF pathways.

Through cell communication analysis, we found that monocytes interact closely with other cells (**Figure 4A**) and played a vital role in multiple signaling pathways, including COLLAGEN, GALECTIN, LAMININ, MIF, MK, ADGRE5, CD46, SEMA4, TNF, ALCAM, CD6 and other signaling pathways (**Supplementary Table 3**). Further in-depth study of each signaling pathway verified that monocytes were directly associated with tumor cells only in the MIF and TNF pathways (**Figures 4B** **, C**), in which the most important ligand-receptors were CD74/CD44 and TNF/TNFRSF1B (**Figures 4D** **, E**). Monocytes in other pathways did not interact directly with tumor cells. They were inclined to indirectly associated with tumor cells through other cells (such as macrophages, T cells, fibroblasts, etc.). This suggests that the changes in the number and function of monocytes mainly promote the progression of tumors indirectly by affecting other cells closely related to tumors, and the direct interaction with tumor cells is a secondary means for monocytes to affect the progression of tumors.

**Figure 5:**
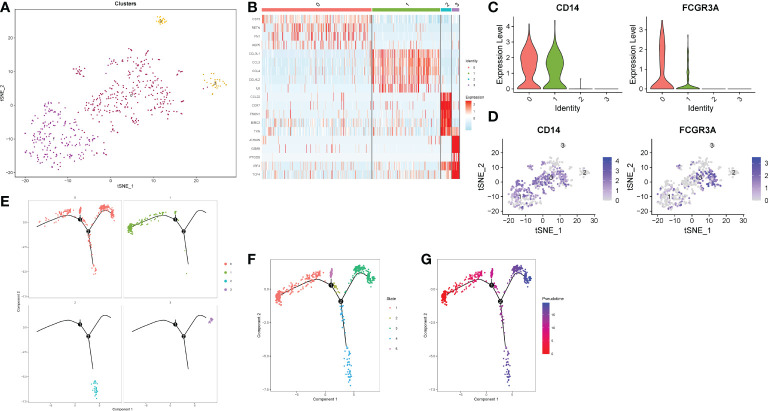
Reclassification of monocyte subsets. **(A)** TSNE dimensionality reduction visualization map of monocyte subsets. **(B)** Heatmap of differential genes in each subgroup. **(C)** A violin plot of normalized expression levels of CD14 and CD16 genes in monocyte subsets. **(D)** Normalized expression level distribution of CD14 and CD16 genes in monocyte subsets. **(E, F)** Distribution of individual cells on the trajectory. **(G)** Maturity of trajectory development in quasi-sequential analysis.

In order to further explore the type of monocytes infiltrating in the gastric cancer microenvironment. We extracted monocyte subsets for subgroup reclassification. Finally, 568 cells and 24159 genes were extracted. After normalizing monocyte subsets again, searching for hypervariable genes, dimensionality reduction and clustering, we identified 4 cell subsets (**Figures 5A** **, B**). Monocytes were usually divided into classical monocytes (CD14 + + CD16-), non-classical monocytes (CD14-CD16 + +) and intermediate monocytes (CD14 + + CD16 +) according to the expression of CD14 and CD16 genes (33). **Figures 5C** **, D** showed the normalized expression levels of CD14 and CD16 in monocyte subsets. The results showed that the infiltrating cells in gastric cancer microenvironment were mainly intermediate monocytes (cluster1, clusters0). In addition, we also found that two monocyte subsets hardly expressed CD14 and CD16 (cluster2, clusters3). Further quasi-timing analysis of monocyte subsets showed that there were two decisive branching points in the development trajectory of monocyte subsets. After determining the starting point of the trajectory by biological significance and statistical methods, we found that most of the cells in cluster0, cluster02 and cluster3 were more mature, while the differentiation degree of cluster1 was relatively naive compared with other subgroups (**Figures 5E** **–G**).

### Acquisition of potential carcinogenic genes from monocytes and establishment of LASSO model

**Figure 6:**
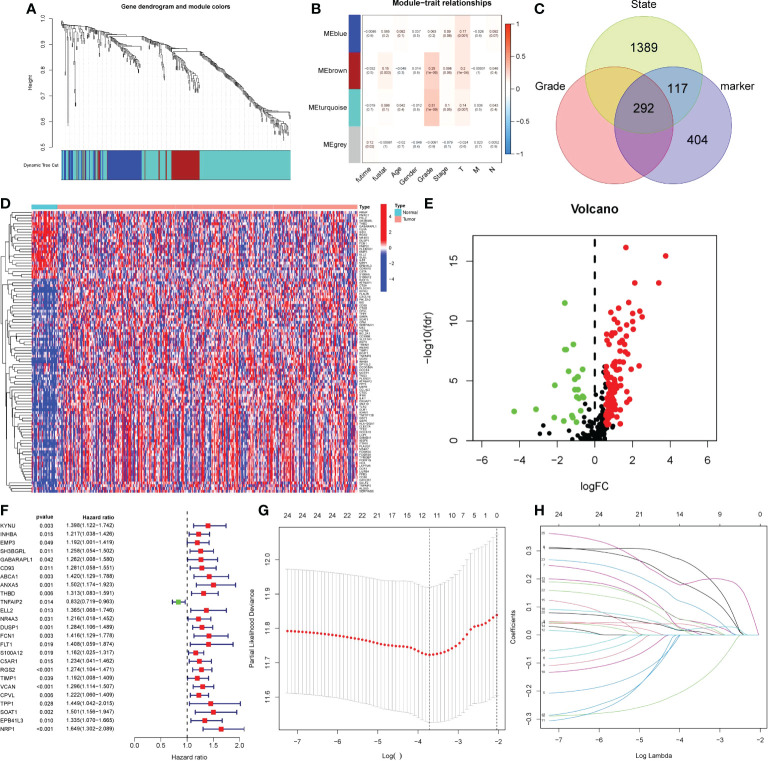
Obtaining monocytic oncogenes and building prognostic models. **(A-B)** Using WGCNA to search for grade-related genes in TCGA. **(A)** Genes were clustered using dissimilarity between genes in the topology matrix. The trees were cut into four modules (the minimum number of genes in each module is 30) by dynamic cutting method. **(B)** Correlation maps of four modules with clinicopathological features. P< 0.05 was statistically significant. **(C)** Venn diagram, three circle distribution represented state related genes, grade related genes and monocyte marker genes. **(D-E)** 135 DEGs between tumor patients and non-tumor patients. Red represented up-regulated gene expression, while green and blue represented down-regulated gene expression. **(F)** The forest map of 25 prognostic related genes was screened by single factor COX analysis. The risk ratio was the correlation between gene expression and prognosis of patients. The greater the risk ratio, the stronger the correlation. When the risk ratio was greater than 1, gene expression was negatively correlated with prognosis, and vice versa. **(G-H)** LASSO regression was used to establish prognostic risk score formula. LASSON regression punished all variables. The coordinates at the lowest point of the red line in panel G were the number of independent variables that had great influence on the dependent variable. Each curve in panel H represented the change trajectory of each independent variable coefficient.

**Figure 7:**
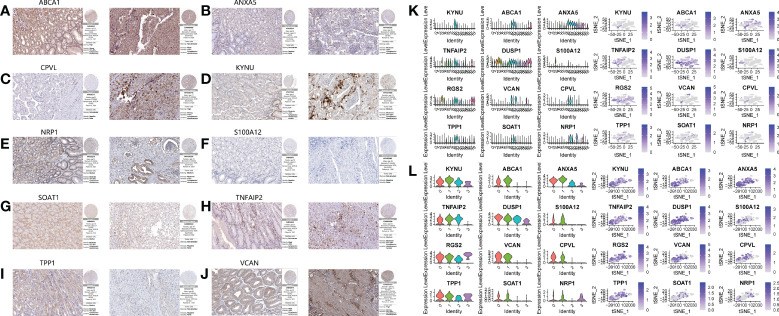
Immunohistochemical results and single cell expression of prognostic model genes. **A–J.** Immunohistochemical results of normal tissues and gastric cancer tissues on HPA website. **(A)** ABCA1. **(B)** ANXA5. **(C)** CPVL. **(D)** KYNU. **(E)** NRP1. **(F)** S100A12. **(G)** SOAT1. **(H)** TNFAIP2. **(I)** TPP1. **(J)** VCAN. **(K, L)** The expression of 12 genes in the primary cluster and subclusters.

We performed WCGNA on the TCGA cohort (**Figure 6A**). The results showed that MEbrown and MEturquoise modules were associated with the patient ‘s Grade (**Figure 6B**). We extracted genes from the corresponding modules and intersected them with monocyte marker genes and cell differentiation-related genes obtained by pseudotime analysis. Finally, 292 genes were obtained (**Figure 6C**). These 272 genes are not only uniquely expressed in monocytes, but also closely related to cell development and differentiation. They control the function, morphology and transformation of monocytes to tumor-associated macrophages, and may be potential oncogenes. Therefore, we speculate that it may have a good effect in predicting the prognosis of gastric cancer patients. Following differential analysis, it was shown that 135 genes were differentially expressed between tumor and normal patients (**Figures 6D** **–E**). Single-factor COX regression analysis eventually selected 25 prognostic-related genes (**Figure 6F**), which were included in the LASSO regression, and finally constructed a risk score formula composed of 12 genes (**Figures 6G** **, H**). The HPA database immunohistochemistry results revealed that 12 genes were overexpressed in human gastric cancer tissues when compared to normal gastric tissues (**Figures 7A** **–L**). The risk scoring formula was as follows:


Risk score=KYNU*0.251+ABCA1*0.015+ANXA5*0.171+DUSP1*0.050+S100A12*0.049+RGS2*0.021+VCAN*0.014+CPVL*0.035+TPP1*0.002+SOAT1*0.107+NRP1*0.187−TNFAIP2*0.183

### Validation of risk scoring formula

**Figure 8:**
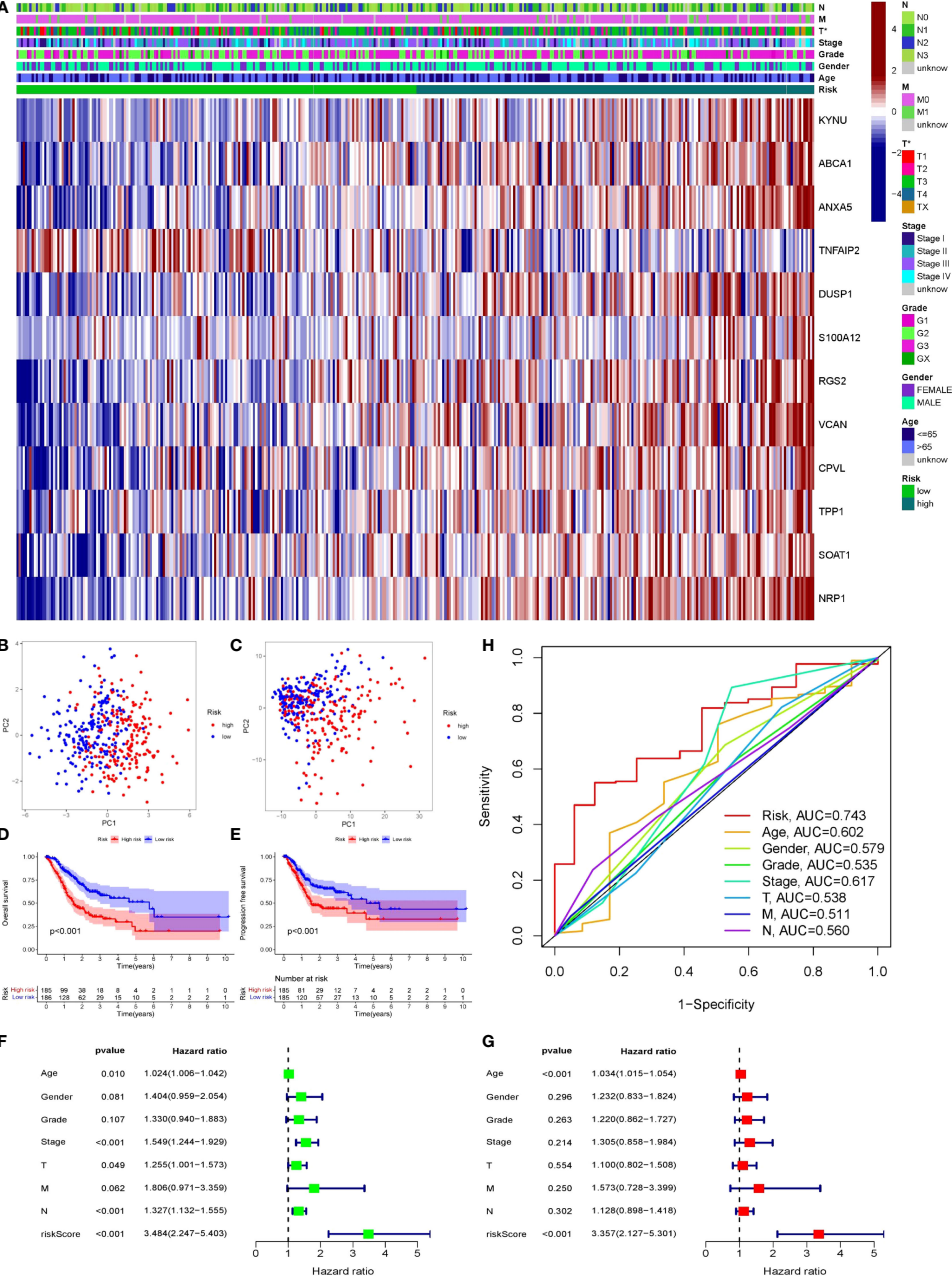
Validation of prognostic models through multiple ways. **(A)** The expression of 12 genes in two groups of patients. Light green represented low-risk group, dark green represented high-risk group. **(B, C)** PCA was performed on two groups of patients. Blue dots represented low risk group and red dots represented high risk group. **(D, E)** Survival analysis of OS and PFS in the two risk groups was conducted based on K-M method. The line represented the survival curve, the area around the curve was the confidence interval, the blue curve was the low-risk group, the red curve was the high-risk group, and the lower axis of the image is the number of surviving patients in the year. **(F, G)** Single-factor and multi-factor COX regression analysis of risk score and clinical pathological characteristics showed the outcome in the form of risk ratio and its 95% confidence interval. **(F)** Forest map of univariate COX regression analysis. **(G)** Forest map of multivariate COX analysis. **(H)** ROC curve of risk score and clinicopathological features, AUC was the area under the curve.

Patients’ prognoses were predicted by their risk score. Patients with a high score had a considerably worse prognosis than those with a low score. We classified patients from the TCGA cohort into high- and low-risk groups using the risk score method. The risk heatmap depicted the expression of 12 genes in patients classified as high or low risk (**Figure 8A**). PCA indicated that the difference between the two groups was obvious, and the prognosis model had a large degree of differentiation (**Figures 8B** **, C**). Patients’ overall survival and progression-free survival were studied. Patients in the high-risk group had a significantly worse prognosis than those in the low-risk group (P<0.001). (**Figures 8D** **, E**). Then we looked at how well clinicopathological characteristics (such as gender, age, TNM stage, Stage, Grade, and risk score) might predict patient prognosis. Age, Stage, T, and N were all found to be adversely connected with patient prognosis in univariate analysis, however their connection intensity was much lower than that of risk score (HR 3.48; p<0.001). (**Figure 8F**). Only risk score (HR 3.36; p<0.001) and age were found to be negatively linked with patient outcome in multivariate analysis. The risk score’s correlation intensity was much higher than that of age. There was no discernible link between clinicopathological characteristics and outcome (**Figure 8G**). Finally, the ROC curve indicated the capacity of the risk score and clinicopathological parameters to predict the prognosis of patients with gastric cancer (**Figure 8H**). The risk score exhibits greater accuracy and stability (AUC = 0.743) when compared to using clinicopathological parameters to predict patient prognosis.

### Drawing and verification of nomogram

**Figure 9:**
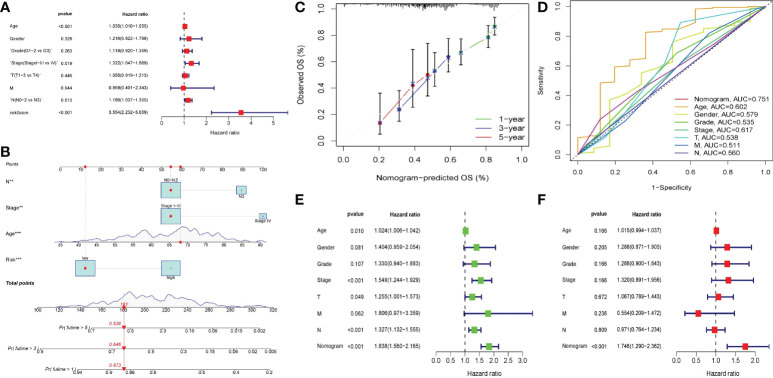
Nomogram of clinical factors and risk scores. **(A)** Forest map of multivariate COX regression of clinicopathological factors after variable transformation. **(B)** The nomogram in which lymph node status (N0-N2 vs N3), staging (Stage I-III vs Stage IV), age and risk score was included to predict 1,3,5-year survival in patients with gastric cancer. The upper part of the line chart was the score of a single factor, and the lower part was the calculation of the total score and the corresponding expected survival rate. **(C)** The calibration diagram of nomogram. The closer the calibration curve in 1,3,5 years was to the diagonal, the more accurate the line diagram was. **(D)** ROC curve of the nomogram. **(E, F)** The univariate and multivariate COX regression analysis of nomogram and clinicopathological features. The outcome in the form of risk ratio and 95% confidence interval. **(E)** Forest map of univariate COX regression analysis. **(F)** Forest map of multivariate COX regression analysis.

We believed that clinicopathological parameters still had some significance for patient prognosis based on the results of prior independent prognostic analyses. Therefore, we established a nomogram through combining clinical pathological indicators with great prognostic significance and risk score, aiming to obtain better prediction methods. We transformed the variables of clinical pathological features, and then analyzed the relationship between them and prognosis. After variable transformation, multivariate COX regression analysis revealed that N, Stage, Age, and Risk were substantially linked with patient prognosis (**Figure 9A**). Based on these three indicators, we build a nomogram (**Figure 9B**). The 1 year, 3 year, and 5 year calibration curves were all quite close to the ideal curve, suggesting that the line chart has a good prediction effect (**Figure 9C**). The nomogram exhibited a better prediction capacity and was more dependable than the risk score (AUC = 0.751), according to the ROC curve (**Figure 9D**). The nomogram was strongly negatively connected with the prognosis in single-factor and multi-factor prognostic analyses, implying that the nomogram had good prognostic prediction capacity (**Figures 9E** **, F**). Since GEO samples as external validation set only contained survival time and survival status, no TNM and age. Therefore, we divided the TCGA queue according to 7:3 and extract the latter as the test set of the nomogram. ROC analysis showed that the nomogram still had a good predictive effect in the test set (AUC = 0.777), and the predictive ability was significantly better than other clinical indicators (**Supplementary Figure 1**).

### Clinicopathological characteristics and immunological infiltration differ amongst patients with different risk scores

**Figure 10:**
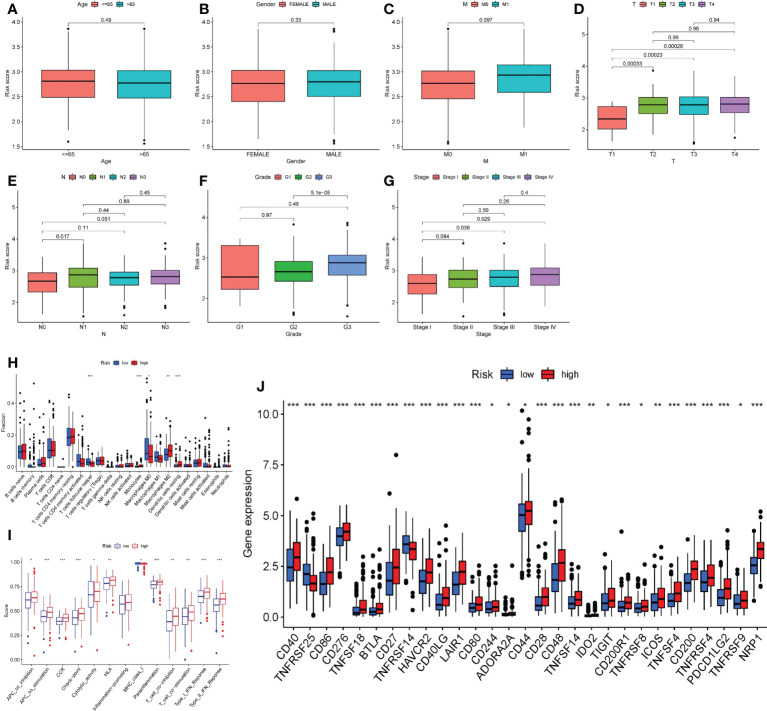
Risk score and clinicopathological features, immune microenvironment. **A–G.** According to the risk score, the patients were divided into high-risk group and low-risk group. Whether the clinicopathological features of the two groups were different was analyzed. **(A)** Age. **(B)** Gender. **(C)** Bloodline transfer(M). **(D)** Primary tumor (T). **(E)** Lymph node metastasis (N). **(F)** Tumor differentiation (Grade). **(G)** Stage. **(H)** The immune cell infiltration of patients in two groups. **(I)** The immune cell function of patients in two groups. **(J)** The immune checkpoint expression of patients in two groups. *P<0.05, **P<0.01, ***P<0.001.

The association between clinicopathological characteristics and risk score was investigated. The results indicated that age, gender, and the occurrence of hematogenous metastasis (M) did not affect the risk score of patients (**Figures 10A** **–C**), while the primary tumor (T), lymph node metastasis (N), tumor differentiation grade), and tumor stage may lead to different risk scores. Specifically, patients with T1 had lower risk ratings than patients with T2 - T4, patients with N0 had lower risk scores than patients with N1 and N3, patients with G1 had lower risk scores than patients with G3, and patients with Stage I had lower risk scores than patients with Stage III-IV (**Figures 10D** **–G**).

After that, we focused on the immune infiltration in patients from various risk groups. The high-risk group had much more M2 macrophages, dendritic cells resting cells, and monocytes than the low-risk group, whereas the infiltration of T cells, follicular helper cells, and M0 macrophages was significantly lower (**Figure 10H**). Furthermore, patients in the high-risk group had a distinct immune function than those in the low-risk group. Many complex immune function changes were observed, including antigen presenting ability and T cell function activation and inhibition, MHC class I function reduction, and type I and type II interferon response enhancement (**Figure 10I**), indicating the tumor immune microenvironment’s complex mechanism. Finally, we looked at the expression of immunological checkpoints in high-risk patients and discovered that a range of immune checkpoints, including as PDCD1LG2, ICOS, CD28, CD40, and others, were strongly expressed in these patients (**Figure 10J**).

### Mutation of 12 genes and response of patients with different risk groups to drug treatment

**Figure 11:**
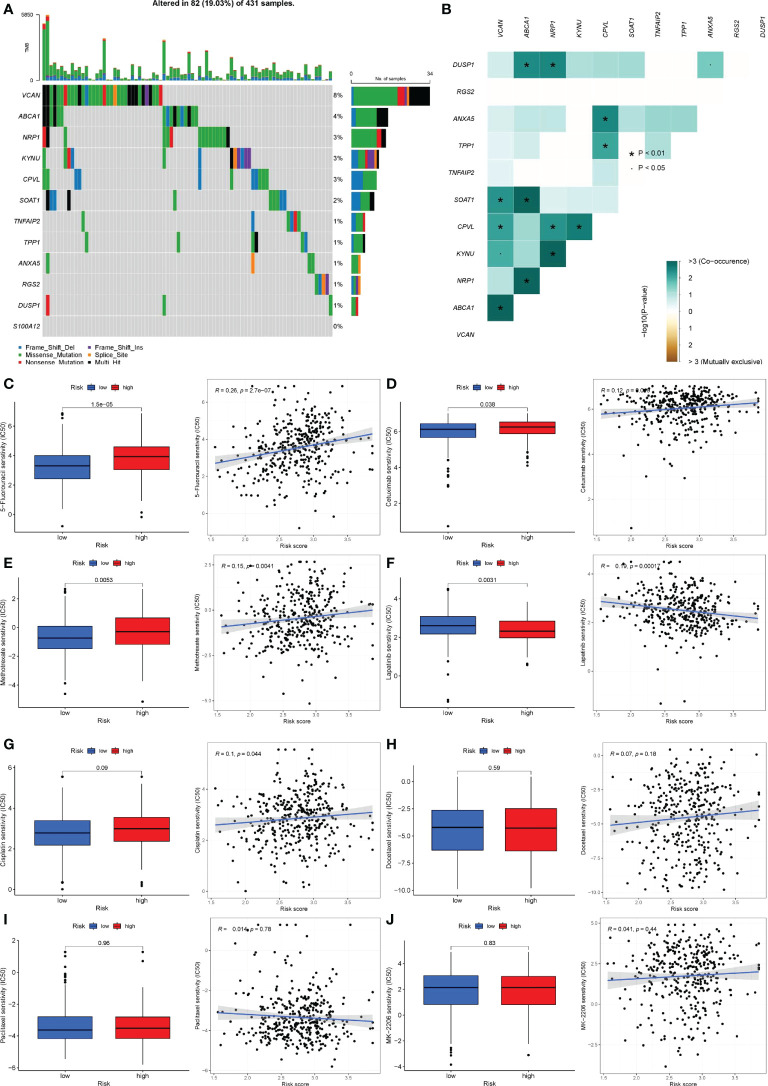
Gene mutation and drug sensitivity analysis. **(A)** The mutation frequency and type of 12 genes. The longitudinal axis was 12 genes, and the transverse axis was the sample with 12 gene mutations. Green was missense mutation, black was multi-site mutation, blue was frameshift deletion mutation, red was nonsense mutation, purple was frameshift insertion mutation, yellow was splice site mutation. **(B)** Co-mutations or co-inhibitions of 11 mutated genes. Light green represented co-mutation; brown represented co-inhibition. P< 0.05, *P< 0.01. **(C-J)** Differences in therapeutic sensitivity of various common gastric cancer drugs to the two risk groups. **(C)** 5-Fluorouracil. **(D)** Cetuximab. **(E)** Methotrexate. **(F)** Lapatinib. **(G)** Cisplatin. **(H)** Docetaxel. **(I)** Taxol. **(J)** MK-2206.

All the 12 genes except S100A12 were mutated to varying degrees. VCAN has the highest mutation frequency of the 11 genes, reaching 8%. ABCA1 was next, with a mutation frequency of 4%. The most prevalent form of mutation was a missense mutation, followed by a multi-hit mutation (**Figure 11A**). In addition, there was a significant co-mutation relationship between genes. Mutations in VCAN could promote mutations in SOAT1, CPVL, KYNU and ABCA1 (**Figure 11B**).

Given that pharmacological therapy remains a critical component of gastric cancer treatment, we assessed the efficacy of commonly used therapeutic agents in patients with gastric cancer who were assigned to different risk groups. 5-Fluorouracil, Cetuximab, and Methotrexate all had a high sensitivity to Lapatinib in patients at high risk (**Figures 11C** **–E**), but individuals at low risk had a higher sensitivity to Lapatinib (**Figure 11F**). There was no statistically significant difference in the sensitivity of cisplatin, docetaxel, paclitaxel, MK-2206, and other medications used to treat patients in various risk groups (**Figures 11G** **–J**). Additionally, we examined the immunotherapy sensitivity of patients in various risk groups. The violin chart indicated that patients in high-risk categories faced a higher risk of immunotherapy, implying that these patients faced a greater likelihood of immunological escape, resulting in immunotherapy failure (**Figure 11K**).

### Enrichment analysis and PPI of patients’ DEGs in different risk groups

**Figure 12:**
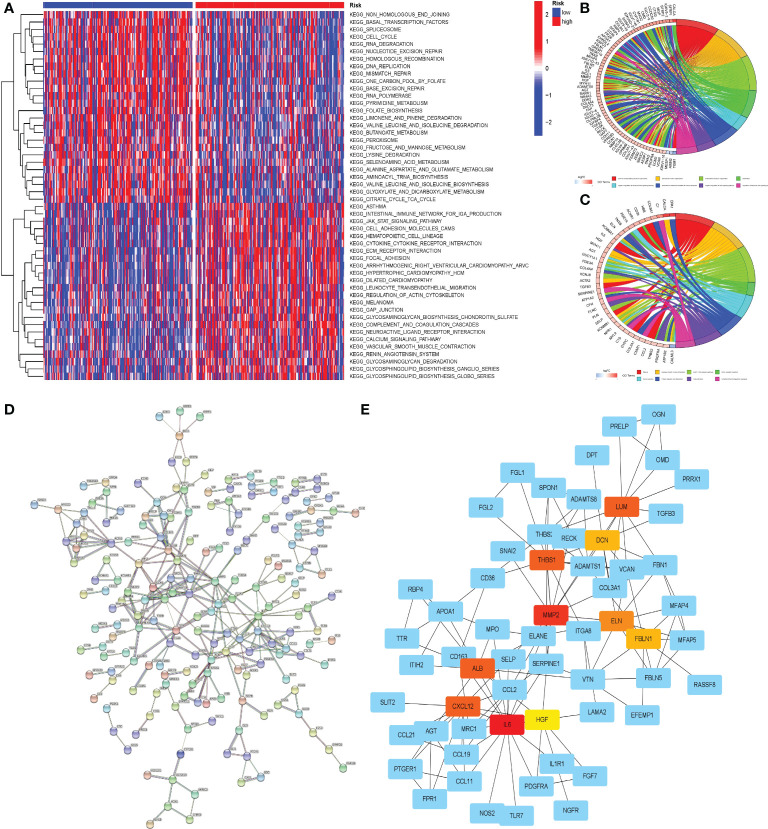
Enrichment analysis and PPI of DEGs between the two groups of patients. **(A)** GSVA of the two groups. **(B)** GO enrichment analysis of DEGs in two groups of patients. **(C)** KEGG enrichment analysis of DEGs in two groups of patients. On the horizontal axis, red represented the high-risk group and blue represented the low-risk group. **(D)** PPI of DEGs on STRING online website. **(E)** Cytoscape software was used to analyze PPI results and visualize core genes. Non-blue represented the core gene in the network.

We were able to clearly see differences in pathways between patients in different risk groups using GSVA analysis. Pathway heatmap showed that the functions of RNA degradation, nucleotide excision repair, non-homologous end joining and cell cycle regulation in patients with high-risk group were significantly down-regulated, while the functions of Jak-Stat signal transduction, cell adhesion, ECM receptor interaction and calcium signaling pathway were up-regulated (**Figure 12A**).

To gain a better understanding of the disparities between patients in various risk groups, we selected the 423 DEGs between the two groups of patients, which would be analyzed through GO and KEGG enrichment. The results of the GO enrichment analysis indicated that the functional differences between high and low risk groups were primarily represented in the extracellular matrix, immunological medium formation, and the Wnt signaling pathway (**Figure 12B**). KEGG enrichment analysis revealed substantial differences in the cGMP PKG signaling pathway, focal adhesion, and ECM receptor impact between the two groups (**Figure 12C**).

PPI analysis was convenient for us to find the core genes in numerous DEGs of the patients in two groups. Through the functions of analysis and visualization in STRING and Cytoscape, we found that LUM, DCN, THBS1, MMP2, ELN, FBLN1, ALB, CXCL12, IL6, HGF and other genes were at the heart of the DEGs network. A complicated network existed between core genes and other DEGs, which may have a significant effect in the progression of gastric cancer (**Figures 12D** **, E**).

## Discussion

As early as 1979, L K Trejdosiewicz et al. pointed out that peripheral blood mononuclear cells can promote the survival of gastric cancer cells (34). In recent years, with the in-depth study of immune microenvironment, people have a more profound understanding of the role of monocytes in the process of gastric cancer. As an important immune cell in the human immune system, monocytes are released from bone marrow into peripheral blood and transported to the whole body with blood, serving as a circulating sensor to respond to environmental changes and diseases (35). Once abnormal changes in the body are monitored, monocytes can directly play an innate immune role, directly kill foreign pathogens and abnormal cells using their phagocytosis, but also further differentiate into macrophages and dendritic cells, and participate in higher adaptive immunity (36). However, monocytes in immune microenvironment play a completely opposite role. Under hypoxic conditions, tumor cells recruit monocytes to infiltrate tumor tissues and modify their functions by releasing tumor-derived factor (TDF). Monocyte chemoattractant protein 1 (MCP-1, also known as CCL2) is one of the most important chemokines (37). Under the action of various TDFs, monocytes gradually lost their anti-tumor ability and had a direct inhibitory effect on other anti-tumor cells. In addition, tumor cells promote the transformation of monocytes to M2 macrophages and inhibit the transformation to M1 macrophages through CSF1 signaling pathway. Therefore, a large number of evidences have proved the important role of monocytes in tumor progression, providing theoretical support for our research.

The results of single cell sequencing data revealed the mechanism of monocyte in the process of gastric cancer. The results of cell subgroups difference analysis showed that the infiltration of monocytes in tumor tissues and normal tissues was significantly different, and monocytes were the key cell subgroups in the immune microenvironment of gastric cancer. Pseudotime analysis suggested that the infiltrated monocytes in tumor tissues were at a low differentiation level, which was consistent with our cognition. Cell communication revealed the cell interaction network in the immune microenvironment of gastric cancer. Monocytes were more likely to indirectly interact with tumor cells through T cells, NK cells, and tumor-related fibroblasts. Direct association with tumor cells was a secondary form of monocyte-mediated tumor promotion. This also suggested that monocytes in the immune microenvironment could be used as targets for immunotherapy. Targeted therapy against monocytes not only inhibited the tumor-promoting effect of monocytes, but also inhibits the ‘umbrella’ of tumor cells by disrupting cellular communication networks in the immune microenvironment.

Our prognostic model included 12 genes that were involved in monocyte differentiation. The product of KYNU is a hydrolytic enzyme involved in tryptophan metabolism that contributes to the synthesis of NAD + cofactors through the canine uridine pathway and is associated with a variety of cardiovascular diseases, inflammation and tumors (38–40). ABCA1 encodes a lipid transporter that facilitates the transfer of phospholipids (PL) and free (unesterified) cholesterol (FC) to extracellular apoA-I and related proteins (41, 42). The ANXA5-encoded protein is a single-chain non-glycosylated protein. Extracellular Anxa5 can bind to the outer membrane of plasma membranes externalized with PS, dying cells, and live leukocytes, where it plays a critical role in hemostasis, apoptosis, and phagocytosis (43–45). TNFAIP2 is highly expressed in monocytes and bladder cells, and participates in NF-κB, Wnt/β-catenin and other signaling pathways in a variety of tumors to regulate inflammation, vascular proliferation, cell proliferation, adhesion and migration (46–48). However, our study discovered that TNFAIP2 expression was positively linked with prognosis, implying that it performs an opposing effect in individuals with gastric cancer. DUSP1 is highly expressed in human paracancerous tissues and overexpression of MAPK pathway induces apatinib resistance in gastric cancer (49). S100A12 belongs to the S100 family, and its protein products are of great significance in anti-infection, tumor cell proliferation, migration and other processes (50–52). RGS2 is a multifunctional RGS protein that effectively interferes with signal transduction by coupling with Gqα receptors, thereby regulating multiple G protein-linked signaling pathways (53). VCAN is a chondroitin sulfate proteoglycan that affects cell adhesion, proliferation, migration and angiogenesis, and also participates in multiple pathological processes such as nervous system and circulatory system (54). CPVL may promote human tumor progression by inhibiting STAT1 pathway through interacting with BTK/p300 axis (55). TPP1 is a component of telomere shelterin and plays a key role in telomere protein complex assembly and telomerase recruitment and regulation (56). SOAT1 is involved in the metabolism of cholesterol in human body. Excess cholesterol can be transformed to inert cholesterol esters *via* sterol-O acyltransferase 1 (SOAT1, also called ACAT1) and SOAT2 (also called ACAT2) (57). NRP1 is a cell surface receptor that is involved in a variety of biological activities, including angiogenesis, immunological response, and regulation of vascular permeability. It has been linked to enhanced cancer progression (58). The fundamental roles of these gene coding products are largely compatible with our findings, indicating that they play a critical role in the progression of gastric cancer.

In this study, patients were classified as high or low risk using the risk score formula, and a range of analyses were performed to demonstrate that the risk score formula has significant guiding importance in clinical practice. The risk score was associated with the patient’s age, primary tumor (T), lymph node metastasis (N), tumor differentiation (Grade), and tumor stage (Stage), but not with the patient’s gender or differentiation (Grade). Therefore, the risk score and prognosis of patients could be preliminarily estimated according to the patient ‘ s age and T, N, M, Stage. Since drug therapy is still the mainstay of gastric cancer treatment, we compared the therapeutic sensitivity of common drugs for gastric cancer to two risk groups to guide the clinical treatment of patients. Our study’s medications had been found to be beneficial in the treatment of stomach cancer, including traditional chemotherapy drugs and targeted drugs (3, 59). According to the results of the study, we believed that 5-Fluorouracil (60), Cetuximab (61), Methotrexate (62) were a better choice in the treatment of patients with high risk score. In the treatment of patients with low-risk score, Lapatinib (63) might achieve better efficacy. Cisplatin (64), Docetaxel (65), Paclitaxel (66) and other agents had no discernible difference in treatment sensitivity between patients with high/low risk scores, and hence risk scores should not be used in selection. This analysis was based on the GDSC website and the ‘pRRophetic’ package. However, the authors of the ‘pRRophetic’ package did not update this package since 2016, which leaded to the new therapies and drugs emerged since 2016, could not be included in the drug susceptibility analysis. It also made our research incomplete. Further studies were needed to confirm the sensitivity evaluation of other common new gastric cancer drugs.

Tumor microenvironment is the accelerator of tumor progression and the barrier of tumor treatment. A variety of immune cells, stromal cells and their secreted cytokines support and interact with each other, leading to the failure of tumor treatment. M2 macrophages, dendritic cells, resting cells, and monocytes increased in the high-risk group, but T cells, follicular helper cells, and M0 macrophages declined. M2 macrophages do not have proinflammatory ability, but inhibit tumor immunity by producing a variety of immunosuppressive factors such as interleukin 10. Dendritic cells also have no antigen presenting capacity, usually involved in the maintenance of immune tolerance (67, 68). Monocytes, as mentioned earlier, are important immune cells that maintain the tumor microenvironment. T cell follicular helper cells are important anti-tumor cells, which can develop or support the recruitment site ELS of CD8 + T cells, NK cells and macrophages to mediate anti-tumor immunity. In addition, Tfh can support B cell anti-tumor antibody response (69). M0 macrophages are unpolarized macrophages, which can be polarized into M1 macrophages with pro-inflammatory effect and M2 macrophages with tumor-promoting effect. M0 macrophages are largely transformed into M2 macrophages in the immune microenvironment, so the number might decrease accordingly (70, 71). In summary, the alteration of immune cell infiltration in the tumor microenvironment in patients classified as high-risk promotes the development of tumor immune tolerance.

Immune checkpoint proteins regulate the immune response in order to preserve self-tolerance and avoid an inflammatory response that is excessive. The high expression of immune checkpoints often leads to the inhibition of normal immune response, which is conducive to tumor progression and metastasis (72). Immune checkpoints such as PDCD1LG2, ICOS, CD28, and CD40 were found to be substantially expressed in the high-risk group, indicating that the tumor microenvironment of the high-risk group had a strong immunosuppressive effect. Patients in high-risk groups are more likely to fail immunotherapy, according to the results of the immunotherapy risk prediction. Without exception, these findings show that patients in the high-risk group had a bad prognosis.

This study still has limitations. Despite a lot of analysis, our findings still need a lot of research to prove its correctness. Since the genes obtained in our study are mainly expressed on the surface of monocytes, our research provides a new research direction and strong theoretical support for the future research on the co-culture of monocytes with gastric cancer cells and organoids for these 12 genes. In addition, due to the limitation of follow-up, the GEO database contains too little chip data on survival time. Even the larger sample GSM2235556 has only 76 samples. Although previous studies have also included it in the test set to detect the accuracy of the model, the sample size is still insufficient compared to the training set. Therefore, large sample sequencing results are again used to verify the predictive ability of the prognostic model is also necessary.

## Conclusion

In summary, we researched the significance of monocyte infiltration in GC patients, and developed and verified the 12 gene signatures used to predict GC patients. In addition, our prediction model has good accuracy and stability, and can well guide clinical diagnosis and treatment.

## Data availability statement

The original contributions presented in the study are included in the article/**Supplementary Material**. Further inquiries can be directed to the corresponding author.

## Ethics statement

Ethical review and approval was not required for the study on human participants in accordance with the local legislation and institutional requirements. Written informed consent for participation was not required for this study in accordance with the national legislation and the institutional requirements.

## Author contributions

WX and DZ designed the research study. WX, DZ and XH selected and collected the data. WX, WZ, DZ and MZ analyzed the data. WX and XH wrote the manuscript. WX, WZ participated in the statical analysis. WZ, DZ, XH, MZ, and CX provided critical opinions and revised the manuscript. All authors read and approved the final manuscript.

## Funding

This study was supported by Science and Technology Plan of Suzhou City (SKY2021038); and Primary Research & Social Development Plan of Jiangsu Province (BE2018659).

## Conflict of interest

The authors declare that the research was conducted in the absence of any commercial or financial relationships that could be construed as a potential conflict of interest.

## Publisher’s note

All claims expressed in this article are solely those of the authors and do not necessarily represent those of their affiliated organizations, or those of the publisher, the editors and the reviewers. Any product that may be evaluated in this article, or claim that may be made by its manufacturer, is not guaranteed or endorsed by the publisher.

## Supplementary material

Supplementary Figure 1 ROC Analysis Results of the Nomogram in the Internal Training Set.

Supplementary File 1 All R codes.

Supplementary File 2 Proportion of each cell subpopulation in each sample.

Supplementary File 3 All marker genes of each cell.

Supplementary File 4 Abnormally Activated Signaling Pathways Identified by Cell Communication Analysis.
