# Supplementary figures and images for: Significance of monocyte infiltration in patients with gastric cancer: A combined study based on single cell sequencing and TCGA
Source: Front Oncol. 2022 Nov 21;12:1001307. doi: 10.3389/fonc.2022.1001307 (PMC9720400; doi:10.3389/fonc.2022.1001307)

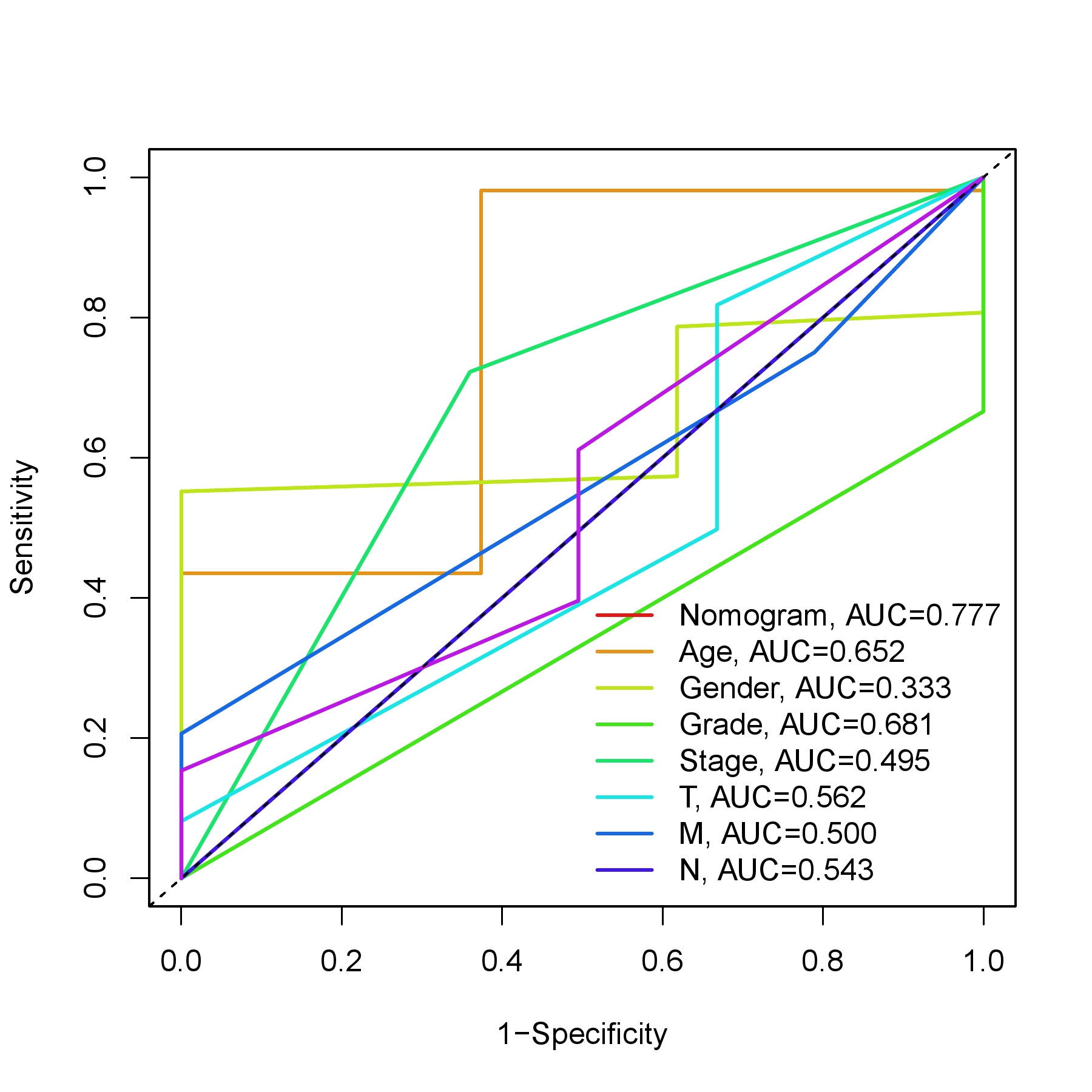

Supplement: Supplementary Figure 1 — ROC Analysis Results of the Nomogram in the Internal Training Set. [file Image_1.jpeg]
